# Comparison of acute non-haemolytic transfusion reactions in female and male patients receiving female or male blood components

**DOI:** 10.1111/j.1365-3148.2007.00802.x

**Published:** 2007-12

**Authors:** S Imoto, N Araki, E Shimada, K Saigo, K Nishimura, Y Nose, Y Bouike, M Hashimoto, H Mito, H Okazaki

**Affiliations:** * Hyogo Red Cross Blood CenterKobe; † The Japanese Red Cross Society, Blood Service Headquarters, Central Blood InstituteTokyo; ‡ Blood Transfusion DivisionKobe, Japan; § Medical Bioinformatics, Kobe University Graduate School of MedicineKobe, Japan

**Keywords:** acute non-haemolytic transfusion reaction, anti-granulocyte antibody, anti-HLA antibody, anti-plasma protein antibody, anti-platelet antibody, TRALI

## Abstract

To study the relationship between antibodies detected in patients’ and/or donors’ sera and the clinical features of acute non-haemolytic transfusion reactions (ANHTRs), and to determine any gender-related difference. ANHTRs range from urticaria to transfusion-related acute lung injury (TRALI). Antibodies to human leukocyte antigen (HLA), granulocytes, platelets, and/or plasma proteins are implicated in some of the ANHTRs. A higher antibody positivity is expected for females than for males. A comparative study of ANHTRs for antibody positivity and their clinical features between females and males for both patients and donors is helpful for characterizing ANHTRs including TRALI more clearly, but such studies are few and outdated. Two hundred and twenty-three ANHTR cases reported by 45 hospitals between October 2000 and July 2005 were analysed. The patients and 196 donors of suspect blood products were screened for antibodies to HLA Class I, HLA Class II, granulocytes, and platelets. The patients were also screened for anti-plasma protein antibodies. The types and severity of ANHTR did not differ significantly between female and male patients. The frequency of the anti-HLA antibodies, but not that of the non-HLA antibodies, was significantly higher in females. Non-HLA antibodies were significantly associated with severe reactions in females. All the TRALI cases had predisposing risk factors for acute lung injury, and 60% of the cases showed anti-leucocyte antibodies. Although the anti-HLA antibodies were detected more frequently in females than males, no significant association of ANHTRs including TRALI with gender, not only for patients, but also for donors, could be shown in this study.

Acute non-haemolytic transfusion reactions (ANHTRs) comprise frequently occurring adverse transfusion events, ranging from mild urticaria to transfusion-related acute lung injury (TRALI) (Combs *et al.*, 2002; Domen & Hoeltge, 2003; Gilstad, 2003; Klein & Anstee, 2005). After the transmission risk of infectious agents has been considerably reduced, ANHTRs have become the most important issues in transfusion safety (Domen & Hoeltge, 2003; Gilstad, 2003; AuBuchon, 2004). In fact, the number of ANHTR cases reported voluntarily by hospitals to the Japanese Red Cross Society Blood Service (JRC) has linearly increased, from 422 in 1995 to 1609 in 2004 (JRC Transfusion Information 9606-25, 0509-91). Antibodies to leucocytes, platelets and/or plasma proteins have been implicated as causative agents of some ANHTRs. TRALI, one of the leading causes of transfusion-related deaths, has been most intensively investigated. However, the reported incidence of TRALI and involvement of antibodies show major discrepancies among related studies owing to inconsistent diagnostic criteria (Silliman *et al.*, 2003; Kleinman *et al.*, 2004; Bux, 2005). Post-transfusion respiratory reaction can be caused not only by TRALI, but also by other entities such as anaphylactic reaction and transfusion-associated circulatory overload (TACO), suggesting that there are borderline cases between TRALI and other ANHTRs (Bux, 2005; Rana *et al.*, 2006). A comparative study of a wide range of ANHTRs, including screening for antibodies will be helpful to characterize TRALI more clearly. However, few such studies have been reported and they tend to be outdated (Barton, 1981; Decary *et al.*, 1984; de Rie *et al.*, 1985). These concerns prompted us to undertake this study.

The Hyogo Red Cross Blood Center (HBC) is a regional blood centre of the JRC, which provides all blood components to hospitals in Hyogo Prefecture with a population of 5·6 million people (4·4% of Japan's population). It is estimated that about 50 000 patients receive transfusions annually. ANHTR cases reported by hospitals to the HBC have increased, from 32 cases in 2000 to 63 cases in 2004. Our objective was to study the relationship between antibodies detected in the patients’ or donors’ sera and the clinical features of ANHTR. For this purpose, we screened antibodies to human leukocyte antigen (HLA) Classes I, and II, granulocytes and platelets in both patients and donors. In addition, patients were screened for antibodies to plasma proteins.

We determined the frequency of antibodies and the relationships between antibodies and ANHTRs, including TRALI. As higher frequency of antibodies can be expected for females, we intended to determine the differences between the results for females and males, not only for patients but also for donors.

## Materials and methods

### Hospitals

Transfusion reactions were reported to the HBC on a voluntary basis by 45 hospitals located in Hyogo Prefecture during the study period from October 2000 to July 2005.

### Patients

Two hundred and twenty-three patients (92 females and 131 males) with ANHTR reported from the 45 hospitals between October 2000 and July 2005 were analysed. Patients’ age, sex, disease, history of transfusion and/or pregnancy and history of previous transfusion reactions as well as clinical features of the transfusion reaction were reported on the JRC reporting form.

### Blood products

Blood components involved in this study were red blood cell concentrates (RCC), platelet concentrates (PC), and fresh-frozen plasma (FFP).

Types of blood donation were 200 or 400 mL of whole blood and apheresis of platelets or plasma. A blood component equivalent to the amount in 200 mL of whole blood has been defined as 1 unit by the JRC. For RCC, red blood cells were suspended in a mannitol–adenine–phosphate solution after centrifugation. All PCs were derived from single donor apheresis. FFP of 1 unit (around 80 mL) or 2 units (around 160 mL) was derived from whole blood, whereas FFP of 5 units (around 450 mL) was derived from single donor apheresis. Regardless of the type of blood component, every bag was derived from a single donor.

### Evaluation of transfusion reactions

ANHTR developing within 24 h after transfusion were evaluated and classified into seven types: Allergy, Anaphylactoid, Anaphylaxis, Febrile, Sepsis-like, TRALI and Dyspnoea unclassified.

‘Allergy’ is the mildest and consists of skin symptoms such as urticaria and pruritus without change in vital signs. ‘Anaphylaxis’ consists of severe hypotension, syncope, or dyspnoea caused by respiratory tract obstruction. ‘Anaphylactoid’ consists of allergic reactions with intermediate severity between allergy and anaphylaxis.

Febrile reactions were classified into ‘Febrile’ or ‘Sepsis-like’. ‘Sepsis-like’ is severe reaction meeting with the criteria of BaCon Study (fever ≥39 °C or ≥2 °C increase, rigors, tachycardia ≥120 b min^−1^ or ≥40 b min^−1^ increase or change in systolic blood pressure ≥30 mmHg rise or drop) (Roth *et al.*, 2001) with negative bacterial culture both in patients’ blood and in suspected blood components. Not so severe febrile reactions were classified into ‘Febrile’.

Post-transfusion respiratory reactions not typical to anaphylaxis were further evaluated based on chest radiographic and clinical data. Pulmonary oedema without signs of circulatory overload was classified into TRALI. Pulmonary oedema should be bilateral, except for patients whose functional lung was unilateral. Otherwise, the post-transfusion respiratory reactions were classified into Dyspnoea unclassified.

Accordingly, Allergy, Anaphylactoid and Febrile were considered as non-severe reactions, whereas Anaphylaxis, Sepsis-like and TRALI were considered as severe reactions. Severity was not determined for Dyspnoea unclassified, as this category includes severe and non-severe reactions.

### Screening for antibodies

#### Sample collection from patients and donors

Patients’ samples were obtained from physicians after obtaining informed consent. Donors’ samples were prepared from suspect blood products in the segment tubes obtained from physicians. Simultaneously prepared plasma components were also examined when available and TRALI was suspected. Before antibody detection, donor's plasma sample was defibrinated by the treatment with thrombin and calcium chloride.

#### Antibodies to HLA antigens

The Enzyme-Linked ImmunoSorbent Assay (ELISA) method (Lambda Antigen Tray™ (LAT); One Lambda, Inc., Canoga Park, CA, USA) was used from October 2000 to September 2004 and the Luminex technology (LABScreen PRA®; One Lambda) from October 2004 to July 2005, according to the manufacturers’ instructions. LAT-M was used for screening and when the reaction was positive, specificity of the antibody was determined using another panel antigen tray, LAT-1240. For the Luminex technology, after incubation with colour-coded beads (LABScreen PRA® Class I and Class II), fluorescent emission from the beads was detected and analysed with the Luminex 100™ IS System (Luminex Corporation, Austin, TX).

#### Antibody to granulocyte antigens

The anti-granulocyte antibody was detected with the micro-mixed passive haemagglutination method (MPHA) using extracted granulocyte antigens (EG-MPHA) as previously reported (Araki *et al.*, 1999). Briefly, Mono-Poly Resolving Medium (Dainippon Pharmaceutical Co., Osaka, Japan) was used to isolate granulocytes from heparin-anticoagulated fresh blood from six volunteer donors. More than 90% of the granulocytes consisted of neutrophils. Next, 3 × 10^3^/μL of granulocytes was suspended in saline containing 3% sucrose, left standing for 3 days at 4 °C, and centrifuged at 10 000 ×*g* for 5 min. The supernatant was then placed in a 72-well U-type Terasaki plate (Robbins Scientific Co., Sunnyvale, CA, USA) and left standing overnight at 4 °C. The antigen-coated tray was stored at −80 °C until use. After thawing and rinsing, the test serum was placed in the wells and left standing overnight at 22 °C in a humidified chamber. After washing, anti-human immunoglobulin-labelled sheep blood cells were added and left standing in a humidified chamber for 4 h at 22 °C. The reactivity was detected by observing the distribution pattern of the erythrocytes.

To detect anti-granulocyte antibodies in HLA antibody containing serum, an antigen-coated tray prepared as described above was treated with chloroquine to inactivate HLA Class I antigens as described previously (Araki *et al.*, 1999).

#### Antibodies to platelet antigens

Screening for anti-platelet antibodies was performed with the aid of an anti-Human Platelet Antigen (HPA)-MPHA panel (Olympus, Tokyo, Japan) according to the manufacturer's instructions. The reactivity to extracted platelet antigens was measured by the MPHA (Shibata *et al.*, 1986). For the detection of anti-platelet antibodies in HLA antibody positive serum, the panel plate was treated with chloroquine to inactivate HLA Class I antigens in accordance with the manufacturer's instructions.

#### Antibodies to plasma protein and screening for serum protein deficiency

Antibodies to IgA, C4, C9, haptoglobin, ceruloplasmin, alpha2 macroglobulin and beta2 glycoprotein I were detected by ELISA as described previously (Shimada *et al.*, 2002), and their presence was confirmed by Western blot analysis (Shimada *et al.*, 2002). For screening for serum protein deficiency, each plasma protein concentrations were measured by means of peak-rate nephelometry (Array 360 System, Beckman Coulter K.K., Tokyo, Japan). Proteins with a concentration below the range detectable by nephelometry were measured with a highly sensitive ELISA method (Shimada *et al.*, 2006).

### Statistical analysis

To determine the significance of differences between two groups, we used the χ^2^ test for 2 × 2 comparisons and Fisher's exact test for 2 × 7 comparisons with alpha level equal 0·05 (Tables 2–4). *P* < 0·05 was considered significant. STATA version 8·2 (StataCorp, College Station, TX, USA) was used to calculate all relevant values.

**Table 2:** Reaction types in female and male patients

	Non-severe			Severe				
				
Gender	Allergy	Anaphylactoid	Febrile	Anaphylaxis	Sepsis-like	TRALI	Dyspnoea unclassified	Total
Female	26	21	14	17	2	1	11	92
Male	42	29	27	15	4	4	10	131
Total	68	50	41	32	6	5	21	223

## Results

### Patients’ characteristics

The characteristics of the 223 patients included in this study are shown in Table 1. The gender ratio was similar to the overall ratio for transfusion recipients, 45 : 55 (42 221 females and 51 128 males), according to the annual data of Tokyo Metropolitan Government (Tokyo Metropolitan Government, 2004). The mean ages and age distributions of the patients were similar for females and males. More than 50% of both female and male patients had haematological diseases. The history of previous transfusion and/or pregnancy was recorded for 76 (83%) of the female patients, with 34 (45%) having a history of both. The history of previous transfusion was recorded for 101 (77%) of the male patients.

**Table 1:** Characteristics of Patients with ANHTR

			Diseases*		History of transfusion/pregnancy		
				
Gender	Number	Age Mean (range)	Haematol	Non-haematol	Yes	No	Unknown
Female	92	54·2 (5–94)	51 (55%)	41 (45%)	76	7	9
Male	131	53·0 (0–94)	67 (51%)	64 (49%)	101	17	13

* Diseases: haematol, haematological diseases; non-haematol, diseases other than haematological diseases.

### Features of ANHTRs of patients

The reaction types of the 223 ANHTR patients are shown in Table 2. The distribution of reaction types did not significantly differ between females and males (*P* = 0·526, Fisher's exact test). Eleven of the 223 cases of ANHTRs were attributed to multiple blood components. Of the remaining 212 cases, 49% were attributed to PC, 42% to RCC and 9% to FFP.

### Antibodies detected in patients

**Fig. 1:**
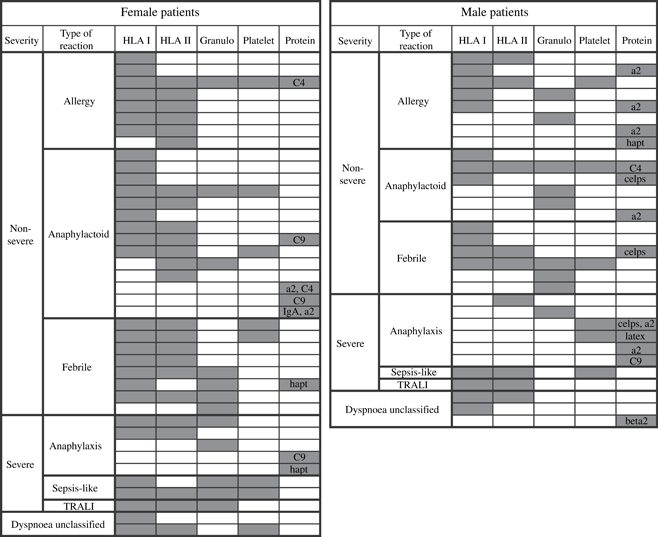
Antibodies detected in female and male patients. Each horizontal row shows the screening result for one patient. Grey cells indicate that the antibody was positive and non-coloured cells indicate that the antibody was negative. HLA-I, anti-HLA Class I antibody; HLA-II, anti- HLA Class II antibody; granulo, anti-granulocyte antibody; platelet, anti-platelet antibody; protein, anti-plasma protein antibody; a2MG, anti-alpha 2 macroglobulin; b2GP, anti-beta2 gl**y**coprotein I; celps, anti-ceruloplasmin; hapt, anti-haptoglobin.

The sera of all the patients were screened for antibodies to HLA Class I, HLA Class II, granulocytes, platelets and plasma proteins. The positivity rate for antibodies was higher for the female patients (43%) than for the male patients (24%) (*P* = 0·00177) (Table 3). The antibody positivity rate associated with Anaphylactoid of the female patients was significantly higher than that of the male patients (*P* = 0·001, Fisher's exact test with Bonferroni correction). ‘Febrile’ and Sepsis-like types showed the same tendency (*P* = 0·086 and *P* = 0·067, respectively). The antibodies detected in each case are shown in Fig. 1. The positivity rate for anti-HLA antibodies for the female patients (36%, 33/92) was significantly higher (*P* = 0·00005) than that for the male patients (13%, 17/131). Of the 33 anti-HLA-antibody-positive female patients, nine (27%) had an antibody to HLA Class I, three (9%) had an antibody to Class II, and 21 (64%) had antibodies to both HLA Classes I and II. For the 17 antibody-positive male patients, the corresponding data were eight (47%), one (6%) and eight (47%).

**Table 3:** Reaction types and incidence of antibodies in female and male patients

	Non-severe			Severe				
				
Gender	Allergy	Anaphylactoid	Febrile	Anaphylaxis	Sepsis-like	TRALI	Dyspnoea unclassified	Total
Female	30·8% (8/26)	66·7% (14/21)	57·1% (8/14)	29·4% (5/17)	100% (2/2)	100% (1/1)	18·2% (2/11)	43·5% (40/92)
Male	19·0% (8/42)	20·7% (6/29)	25·9% (7/27)	40% (6/15)	0% (0/4)	25% (1/4)	30% (3/10)	23·7% (31/131)
Total	23·5% (16/68)	40% (20/50)	36·6% (15/41)	43·8% (14/32)	33·3% (2/6)	40% (2/5)	23·8% (5/21)	31·8% (71/223)

The positivity rates for the anti-granulocyte antibody were 13% (12/92) for the female patients and 7% (9/131) for the male patients (*P* = 0·1202). Those for the anti-platelet antibody were 9% (8/92) for the female patients and 5% (6/131) for the male patients (*P* = 0·2123).

The positivity rates for the anti-plasma protein antibodies were similar between the female and male patients, i.e. 9% (8/92) and 10% (13/131), respectively. Of the 21 anti-plasma protein antibody positive cases, 18 (86%) showed allergic reactions (Allergy, Anaphylactoid or Anaphylaxis). Two of the three anti-haptoglobin-positive patients were haptoglobin deficient, as reported elsewhere (Shimada *et al*., 2006; Shimode *et al.*, 2006). Except for these two cases, none of the anti-plasma protein antibody positive cases showed plasma protein deficiency.

Of the 40 antibody-positive female patients, eight showed severe reactions (five Anaphylaxis, two Sepsis-like and one TRALI). Of these eight patients, seven (87·5%) showed positivity for antibodies other than HLA (non-HLA antibodies: five anti-granulocyte antibody and two anti-plasma protein antibody). However, only 46·7% (14/30) of the patients with non-severe reactions showed such positivity (*P* = 0·0388). For the male patients, no significant difference was observed. Among the 31 antibody-positive male patients, six (75%) of the eight patients with severe reactions and 16 (80%) of the 20 patients with non-severe reactions showed non-HLA antibodies.

### Features of ANHTRs and associated donors

One hundred and ninety-six donors of blood components associated with 134 ANHTR cases were examined. The blood components consisted of 85 RCCs, 57 PCs and 54 FFPs (Table 4). The relatively higher ratio of FFPs was because of the inclusion of patients who were transfused with multiple units of blood components. Of the 196 donors, 67 (34%) were female and 129 (66%) were male. Because the donors’ gender ratio differed depending on the type of blood donation (Blood Products Research Organization, Annual Report 2001–2005), the expected values calculated from the annual donation data were 32% female and 68% male, which are almost similar to the actual data.

**Table 4:** Reaction types and donor's gender of suspect blood component

	Non-severe			Severe				
				
	Allergy	Anaphylactoid	Febrile	Anaphylaxis	Sepsis-like	TRALI	Dyspnea- unclassified	Total
Number of cases	35	35	20	18	5	3	18	134
Suspect	FFP 5	FFP 25	FFP 8	FFP 11		FFP 5		FFP 54
Blood components	RCC 13	RCC 14	RCC 15	RCC 10	RCC 6	RCC 9	RCC 18	RCC 85
	PC 20	PC 15	PC 6	PC 9		PC 1	PC 6	PC 57
Donor's gender								
Female	10	14	14	13	3	5	8	67
Male	28	40	15	17	3	10	16	129
Total	38	54	29	30	6	15	24	196

The distribution of associated reaction types showed no significant difference between the female and male donors (Table 4) (*P* = 0·304, Fisher's exact test). Fifty-one donors were associated with severe reactions (30 Anaphylaxis, 6 Sepsis-like and 15 TRALI), of whom 21 were female (21/67, 31·3%) and 30 were male (23·3%, 30/129) (*P* = 0·221).

### Antibodies detected in donors

**Fig. 2:**
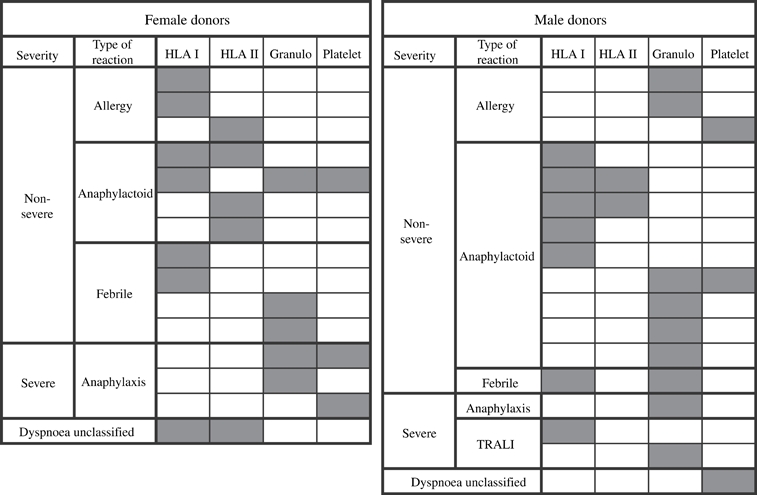
Antibodies detected in female and male donors. Each horizontal row shows the screening result for one donor. Grey cells indicate that the antibody was positive, and non-coloured cells indicate that the antibody was negative. HLA-I, anti-HLA **C**lass I antibody; HLA-II, anti-HLA **C**lass II antibody; granulo, anti-granulocyte antibody; platelet, anti-platelet antibody.

The antibodies detected in the donors are shown in Fig. 2. Of the 196 donors, 32 (16%) donors were antibody-positive, of whom 15 were female (15/67, 22%) and 17 were male (17/129, 13%) (*P* = 0·09799). The positivity rates for antibodies to HLA Class I and/or Class II were 15% (10/67) for the female donors and 5% (7/129) for the male donors (*P* = 0·025). The corresponding results for the anti-granulocyte antibody were 8 and 7% and those for the anti-platelet antibody were 4 and 2%. Although the positivity rates for anti-HLA antibodies were significantly higher for the female donors, those for antibodies to granulocytes and platelets showed no significant difference between the female and male donors.

Concerning severity, three of the 15 (20%) antibody-positive female donors were associated with severe reactions (Anaphylaxis). All three showed positivity for the non-HLA antibodies (two, anti-granulocyte antibody; one, anti-platelet antibody); however, only three of the 11 donors (27·3%) associated with non-severe reactions had non-HLA antibodies (*P* = 0·0241). However, three of the 17 (17·6%) antibody-positive male donors were associated with severe reactions. Two of the three donors (66·7%) associated with severe reactions had non-HLA antibodies, whereas eight of the 13 donors (61·5%) associated with non-severe reactions had non-HLA antibodies. Although the positivity rate for the non-HLA antibodies was significantly higher in the female donors associated with severe reactions than in those associated with non-severe reactions, it was not the case in male donors.

### Analysis of TRALI cases

The characteristics of 26 patients with post-transfusion respiratory reactions not typical to Anaphylaxis were analysed. Chest radiographic data were available for only 15 of the 26 patients. Of these 15 patients, seven showed pulmonary oedema and/or congestive shadow. The remaining eight patients showed no signs of pulmonary oedema and were classified as having Dyspnoea-unclassified. Five patients were classified as having TRALI (Table 5). Although pulmonary oedema was unilateral owing to congenital anomaly of one lung, patient 5 otherwise met the TRALI criteria and was classified as having TRALI. All the five patients showed risk factors for acute lung injury (two cardiac surgery, two pneumonia and one sepsis) before transfusion. Regarding the remaining two patients, TACO was suspected in one, and anaphylactic reaction was considered as more likely for the other. Both patients as well as the 11 patients without available chest radiographic data were classified as having Dyspnoea-unclassified.

**Table 5:** Characteristics of five TRALI cases

Case number	Age	Gender	History of transfusion/pregnancy	Disease	Factors of ALI	State of hypoxaemia	Chest radiography	Patient's antibody	Blood components (donor's gender)	Donor's antibody
Case 1	70	Male	Unknown	Angina pectoris	Yes, bypass surgery	Mechanial ventilation	Bilateral oedema	Negative	7 RCCs (3 f 4 m) 5 FFPs (1 f 4 m)	HLA-I (RCC; male) granulo (RCC; male)
Case 2	89	Female	Yes	Chronic cardiac failure	Yes pneumonia	*p* O2 < 60 mmHg	Bilateral oedema/congestion	HLA-I HLA-II granulo	2 RCCs (1 f 1 m)	Negative*
Case 3	82	Male	No	Chronic lymphoid leukaemia	Yes pneumonia	Mechanial ventilation	Bilateral oedema	Negative	1 RCC (m)	Negative
Case 4	75	Female	Yes	Acute myeloid leukaemia	Yes sepsis	SpO2<90% with reserver bag	Bilateral oedema	Negative	1 RCC (f) 1 PC (m)	Negative
Case 5	11	Male	Yes	Transposition of great artery	Yes cardiac surgery	Mechanial ventilation	Right oedema (left atelectasis)	HLA-I HLA-II	2 FFPs (2 m) 4 RCCs (1 f 3 m)	ND

* Detection of antibodies to HLA **c**lass I, **c**lass II and granulocytes was carried out from the plasma product by flow cytometry. f, female; m, male; ND, not defined.

For the five TRALI patients, the involvement of 23 blood components was suspected; seven components (30·4%) were derived from the female donors and 16 (69·6%) from the male donors (Table 5). Three patients (patients 1, 2 and 5) showed positivity for anti-HLA and/or anti-granulocyte antibodies. For patient 1, seven RCCs and five FFPs were transfused during and after coronary artery bypass graft surgery. The anti-HLA Class I antibody was detected in one RCC, and the anti-granulocyte antibody in another RCC, both of which were derived from the male donors. The cross-match test of lymphocytes of patient 1 and the donor's anti-HLA-positive serum indicated negativity for anti-human immunoglobin-lymphocyte cytotoxicity test (AHG-LCT). Moreover, the antibody specificities determined by LAT-1240 were for A1 and A36, whereas the patient's HLA types were A24/26, B54/62 and Cw1/9. In contrast, the anti-granulocyte antibody was reactive with all the 12 antigen panels.

Antibodies to HLA Classes I and II, and granulocytes were detected in the serum of patients 2, and antibodies to HLA Classes I and II in that of patient 5. No cross-match test between the patient's sera and the donor's lymphocytes could be performed for these two patients.

## Discussion

Our present study showed that the incidences of ANHTR and the distributions of ANHTR by type and severity were similar regardless of the gender of either the patients or the donors. However, most of the patients in this study were transfused previously. To determine whether there is any difference in ANHTR between male patients with no transfusion history and female patients, a more intensive study is necessary.

As expected, the positivity rates for anti-HLA antibodies were significantly higher for females, with 36% for the female patients, 13% for the male patients, 13% for the female donors and 5% for the male donors. These were comparable with those of other reports. Densmore *et al.* reported that the positivity rates for anti-HLA antibodies for female apheresis donors range from 7·8 to 26·3% depending on their parity (Densmore *et al.*, 1999) and Bray *et al.*(2004) showed that anti-HLA antibodies were detected in 22% of blood components by FlowPRA. Uhrynowska and Zupanska (1996) found that the positivity rate for the anti-HLA antibody was 21·3% for patients who undergo frequent platelet transfusion. To the best of our knowledge, however, this is the first comparative study of patients and donors of both sexes. The 13% positivity rate of the male patients was same to that of the female donors, probably owing to previous transfusion for the male patients. Whether 5% positivity rate of the male donors is higher than that of the normal male population, the screening of male donors not associated with ANHTR appears necessary.

However, the positivity rate for the anti-granulocyte antibody did not differ significantly, not only between the male and female patients, but also between the patients and the donors. The positivity rates for the anti-granulocyte antibody were 13% for the female patients, 7% for the male patients, 8% for the female donors and 7% for the male donors. The 13% positivity rate of the female patients was comparable to that reported by Clay *et al.*; 12·6% of postpartum women show granulocyte agglutinating antibodies (Clay *et al.*, 1984). The 7% positivity rate of the male patients was comparable to that of the female donors in our study. However, 7% positivity rate of the male donors was unexpectedly high, because blood donors in Japan should have no transfusion history; thus, a much lower incidence is expected. We used the EG-MPHA method indicated in Materials and Methods. Although the influence of anti-HLA antibodies should be of concern, seven (77·8%) of the nine anti-granulocyte-antibody-positive male donors had neither the anti-HLA nor anti-platelet antibody, as shown in Fig. 2, suggesting that the detected antibodies bind to granulocyte antigens other than HLA. Although anti-leukocyte antibodies have long been implicated in febrile reactions, our results showed no clear association between them.

No significant differences in the positivity rate for anti-platelet antibody, 9% for female patients, 5% for male patients, 4% for female donors and 2% for male donors, were observed between females and males. Although reports on the anti-platelet antibody in transfusion patients are few, Uhrynowska and Zupanska (1996) detected the anti-platelet antibody in 9·6% of sera from platelet-transfused patients using the platelet suspension immunofluorescence test and the monoclonal antibody immunobilization of platelet antigens (MAIPA) technique; this result is comparable to that of our female patients. We used a commercial HPA-MPHA kit indicated in Materials and Methods. Although the influence of anti-HLA antibody should be of concern, all the three anti-platelet-antibody-positive male donors were anti-HLA-negative, as shown in Fig. 2. Interestingly, the anti-platelet antibody appears to be associated with allergic reactions. Although reports of anti-platelet antibody-associated ANHTR other than platelet transfusion refractoriness are few, Morishita *et al.*(2005) reported a case of severe anaphylactic-like reaction (hypotension, chest tightness and nausea) followed by thrombocytopenia after the transfusion of FFP containing an anti-CD36 (NaK^a^) isoantibody, which was detected using the HPA-MPHA kit, the same kit that we used.

In this study, only five of the 26 cases of post-transfusion respiratory reactions could be classified as TRALI. The remaining 21 cases had to be classified as ‘Dyspnoea-unclassified’, because of the absence or inadequacy of chest radiographic data and/or incompatible clinical findings. Table 5 shows that three of the five (60%) TRALI cases, two patients and two donors, showed anti-leukocyte (HLA and/or granulocyte) antibodies. The positivity rate for antibodies of TRALI cases was similar to that reported by the JRC, in which the positivity rate was 54·3% (32·3% for patients and 27·7% for donors) (JRC Transfusion Information 0403-81); however, the positivity rates in the literature, of mostly donors, range from 3·6% (Silliman *et al.*, 2003) to 89% (Popovsky & Moore, 1985). For patient 1 in Table 5, 2 of the 12 suspected donors showed positivity for the anti-HLA or anti-granulocyte antibody. The anti-granulocyte antibody was reactive with all the antigen panels, suggesting reactivity with commonly expressed granulocyte antigen(s); thus, it may be implicated in TRALI. Unexpectedly, both donors were male, in contrast with that in the literature showing that most of donors implicated in TRALI are female and predominantly multiparous (Popovsky & Moore, 1985; Davoren *et al.*, 2003; Kopko *et al.*, 2003; Wallis *et al.*, 2003). Our present study showed no female donor predominance in either TRALI or other ANHTRs. In addition, the positivity rates for antibodies to HLA, granulocytes and/or platelets in the male donors were unexpectedly high. At present, we cannot explain these results accurately. Further study is necessary.

A two-event model has been proposed for the aetiology of TRALI, the first event being the clinical condition of the patients and the second the transfusion of a biological response modifier (Silliman *et al.*, 2005). Our results showed that all the five TRALI cases had risk factors for ALI, such as cardiac surgery and pneumonia, thus supporting the two-event model to some extent.

The standardization of assays also appears necessary. For anti-HLA antibodies, commercially available extracted panel antigens have come into widespread use (Worthington *et al.*, 2001; Insunza *et al.*, 2004; Sato *et al.*, 2005; Mihaylova *et al.*, 2006). For anti-platelet antibody screening, the commercial HPA-MPHA kit described in Materials and Methods has been widely used in Japan with acceptable reproducibility (Shibata *et al.*, 1986; Ohto *et al*., 2004; Morishita *et al.*, 2005, Morita *et al.*, 2006). Although the method is as sensitive as MAIPA, and can be more easily manipulated, large-scale study to compare with standard methods is necessary to make it a standard method worldwide. The screening for the anti-granulocyte antibody is more difficult, because no commercial kit is available yet and in most laboratories the preparation of fresh leukocytes is necessary for each assay (Lucas *et al.*, 2002; Insunza *et al.*, 2004; Stroncek, 2004). Our use of the EG-MPHA method facilitated the screening for the anti-granulocyte antibody because the extracted granulocyte antigens could be frozen and stored for a long period and could be used simply by thawing (Araki *et al.*, 1999; Tanaka *et al.*, 2000; Han *et al.*, 2006). To make it a standard assay, however, a large-scale preparation of panel antigens, as well as a large-scale study to compare with other methods is necessary (Stroncek, 2004).

To summarize, no significant association of ANHTR with gender, not only for patients, but also for donors, was shown in our study. Although antibodies to granulocytes, platelets and HLA may be associated with severe ANHTRs, including TRALI, a large-scale study based on common criteria and using standardized assays is necessary to obtain more detailed information.
